# Bipolar Disorder in a female with CDKL5 Deficiency Disorder: A Case Report

**DOI:** 10.21203/rs.3.rs-4851179/v1

**Published:** 2024-09-10

**Authors:** Jenna Lucash, William Hong, Lindsay Swanson, Kiran Maski, David Urion, Jung Kim, Helen Leonard, Heather Olson

**Affiliations:** Boston Children’s Hospital; Boston Children’s Hospital; Boston Children’s Hospital; Boston Children’s Hospital; Boston Children’s Hospital; Boston Children’s Hospital; Telethon Kids Institute; Boston Children’s Hospital

**Keywords:** CDKL5 Deficiency Disorder, Bipolar Disorder-unspecified, developmental and epileptic encephalopathy, sleep disorders, psychiatric disorders, Neurodevelopmental disorders, Lifespan manifestations of childhood onset conditions, epilepsy

## Abstract

**Background:**

CDKL5 deficiency disorder (CDD) is an early-onset developmental and epileptic encephalopathy. While a subset of individuals is believed to experience comorbid behavioral disorders, none have reported well-defined affective disorders. Though there is a documented association between epilepsy and mood disorders, they may go undetected in the CDD population due to difficulty assessing mood in the presence of severe/profound intellectual disability and disease-related sleep dysregulation. We aimed to identify the clinical characteristics of an individual with CDD who presented with a mood disorder falling outside this expected behavioral phenotype.

**Case Presentation:**

We identified one 22-year-old female with CDD diagnosed with unspecified bipolar disorder at 18 years of age. Family history was noncontributory. At diagnosis, she had fluctuations in mood, characterized by periods of elated affect, increased energy and vocalizations, hypertonia, and insomnia lasting 3–4 days alternating with periods of depressed affect, irritability, hypotonia, and excessive sleep lasting for up to one month. She had experienced frequent mood swings and sleep dysregulation from early childhood, and by early adulthood the duration of “up” and “down” periods fell in the range specified in the DSM-5 bipolar disorder criteria. Trazodone and suvorexant did not alleviate sleep related symptoms. Her epilepsy was well controlled on lamotrigine monotherapy since early childhood. Though lamotrigine treatment has had no psychiatric benefit despite its known mood stabilizing properties, aripiprazole has been effective in reducing severity and frequency of fluctuations between hypomania and depression.

**Conclusions:**

While sleep and behavioral disorders fall within the expected phenotype for CDD, this is the first report of bipolar disorder. Careful attention to patterns of sleep and behavior that may indicate mood cycling in this population is required, particularly in the setting of limited communication and functional abilities.

## Background

CDKL5 Deficiency Disorder (CDD) is a developmental and epileptic encephalopathy caused by loss of function variants in the gene encoding the serine-threonine kinase, cyclin-dependent kinase-like 5. Though CDD has a wide phenotypic spectrum, commonly reported characteristics include infantile onset, often refractory epilepsy, hypotonia, global developmental delay and later intellectual and motor disabilities, and cortical visual impairment (1). Sleep dysfunction is also common in this population, with 86.5% of individuals captured by the International CDKL5 Deficiency Disorder (ICDD) and InterRett Databases reported as having “sleep problems” on parent questionnaires (2–5). The most frequently reported types of sleep disturbance were excessive nighttime waking and excessive daytime napping. Though behavioral comorbidities such as autism spectrum disorder (ASD), attention deficit hyperactivity disorder (ADHD), and non-specific irritability have also been observed in individuals with CDD, there is limited formal data on their frequency and clinical presentation (1, 6).

Further, despite the epidemiological and potential mechanistic association between epilepsy syndromes more generally and mood disorders, no individuals with CDD have been reported in the literature as a having a formal diagnosis with an affective disorder (1, 7). In a survey study of US adults with and without epilepsy, bipolar disorder was reported to occur in adults with epilepsy at a rate of more than double that of those without epilepsy (Propensity ratio 2.11 (95% CI 1.82–2.45)) (8). Variants in another epilepsy gene, *KCNQ2*, have been implicated as potential susceptibility loci for bipolar disorder (9, 10). However, the rates of occurrence of bipolar disorder in individuals with genetic developmental and epileptic encephalopathies broadly is not well described.

We suspect that bipolar and other affective disorders may go undetected in the CDD population due to difficulty assessing mood in the presence of disease-related sleep dysfunction and impairments in expressive communication brought about by severe/profound intellectual disability (ID). However, there have been isolated reports of individuals with CDD displaying symptoms of mood dysregulation within our own cohort and from our colleagues at the other CDKL5 Centers of Excellence that warrant further investigation ((6), unpublished data). As such, we aimed to identify clinical characteristics of individuals with CDD that had presented to our clinic and the other US and Australia CDKL5 Centers of Excellence with mood disorders falling outside the expected phenotypic spectrum. We identified a single individual diagnosed with bipolar disorder, thus meeting these criteria. In describing her history and presentation, we hope to improve awareness and detection of affective disorders in the CDD population

### Case presentation

A now 22-year-old female with CDKL5 deficiency disorder and related ID presented at 18 years of age with a 13-year history of disruptive behaviors, irritability, and irregular sleep in addition to typical features of CDD. DNA sequence analysis of *CDKL5* exons 2–21 from whole blood drawn at age 4 identified a pathogenic frameshift variant (c.1791delC, p.Tyr598Thrfs*18) in exon 12 of the *CDKL5* gene.

### Neurological history:

This individual was born full term via uncomplicated spontaneous vaginal delivery. In the first three months she demonstrated irritability and poor eye contact. At three months of age, she presented with an afebrile generalized tonic-clonic seizure. Head MRI was normal. Despite treatment with phenobarbital, seizures persisted on a daily basis, with a semiology of generalized tonic-clonic and generalized tonic, often progressing to myoclonic jerking of upper and lower extremities.

Her epilepsy was initially refractory to medication but mostly controlled on lamotrigine monotherapy by 4 years of age, with the exception of isolated breakthrough seizures with illness. At age 22 years, she is more than 5 years seizure free and off anti-seizure medications for 8 months. Consistent with diagnosis of CDD, she additionally has cortical visual impairment, profound ID without verbal communication, ASD, hand and vocal stereotypies (touching, mouthing, flapping, buzzing), axial and appendicular hypotonia with related complications (dysphagia and g-tube dependency, kyphoscoliosis, limited mobility), and gastrointestinal dysfunction (chronic constipation, gastro-esophageal reflux, slow gastric emptying).

### Psychiatric Presentation:

Beginning at 5 years of age, she developed episodes of insomnia, near constant stereotypies, and giggling lasting 2 to 3 days, alternating with 24- to 36-hour bouts of hypersomnia, heightened irritability, and increased hypotonia. The pattern was not related to seizures, and her symptoms worsened in severity and duration over the course of the next several years. By 18 years of age, her fluctuations in mood were characterized by periods of elated affect, increased energy and vocalizations, hypertonia, and decreased need for sleep (24–36 hours of continuous waking without detriment to mood and activity level) lasting 3–4 days, alternating with periods of depressed mood, irritability, hypotonia, and excessive sleep (24–48 hours of sleep at a time) lasting for up to one month. Given that she is non-verbal, assessment of affect and mood was made through direct observation and reports from her mother of patient activity (increased stereotypies, rocking, and willingness to engage in therapies and activities versus lethargy, somnolence and social withdrawal during school and therapies), content of vocalizations (giggling versus crying), and facial expressions (excessive grinning versus frowning or flat facial expression). A diagnosis of bipolar disorder - unspecified type was made by psychiatry (JK) based on DSM-5 criteria.

### Pharmaceutical management:

Between the ages of 5 and 18 years, medication trials of citalopram, risperidone, escitalopram, mirtazapine, clomipramine, and aripiprazole for stereotypies, irritability, and screaming episodes were found to be ineffective. Suvorexant, clonidine, trazodone, and quetiapine were also trialed and ineffective for insomnia. While lamotrigine is used for treatment of bipolar disorder, it did not provide clear benefit for mood while she was treated with this medication for epilepsy (maximum dose 500 mg daily).Aripiprazole re-trialed at 18 years (current dose 20 mg PO daily) was initially well tolerated and reduced severity of hypomania and frequency of depressive episodes. However, due to waning effectiveness of aripiprazole over time, mood stabilizers including lithium are being considered.

### Sleep Dysfunction in the Boston Children’s CDKL5 Cohort and the International CDKL5 Disorder Database

Given that extreme sleep dysregulation was an important component of this individual’s presentation leading to her diagnosis with bipolar disorder, and that no other individuals with a formally diagnosed mood disorder were identified from our CDD cohorts, we reviewed the cohort of patients enrolled under the CDKL5 Clinic Study observational protocol at Boston Children’s Hospital (IRB-P00016602) for individuals exhibiting similarly extreme sleep disturbances. Patients were enrolled by informed consent or waiver of consent for use of retrospective data. We excluded individuals under the age of 5 years from this review due to expected sleep dysregulation attributable to immaturity of sleep patterns prior to this age. Of the 77 patients in this cohort, we identified 4 individuals with sleep dysregulation resembling that of the individual described above. Of these patients, only one, a 6-year-old male at time of evaluation, demonstrated alternating periods of insomnia and hypersomnia (up to 24 hours with or without sleep). Three individuals were noted to have periods of hypersomnolence with 17- and 24-hour periods of sleep. In these individuals, however, there is not a clearly established association with a mood disorder. One individual had entire nights without sleep, suspected to be related to gastrointestinal discomfort.

The International CDKL5 Disorder Database has also been collecting questionnaire information from caregivers on sleep disturbances in their children. The section on sleep specifically included a question where respondents were asked if there was anything about their child’s sleeping which they considered unusual or problematic. In those individuals aged 5 years and over (n = 203), 13 caregivers responded to this question by specifically describing a sleep pattern with similarities to those exhibited by the patient in our case study. In seven (six females and one male) they alluded to nights when their child was awake all night (sometimes referred to as an “all night party”) and in another six females this pattern was also present but reported to be followed by a prolonged period of sleeping. These episodes could have occurred in the past but at the time of questionnaire submission ages ranged from 5.7 to 22.3 years with a median of 13.8 years.

## Discussion and Conclusions

While this is the first report of an individual with CDD and a formal diagnosis of bipolar disorder, we believe there is a possibility that bipolar disorder may be going undetected in individuals with CDD displaying similar symptoms due to challenges assessing mood and thought content in individuals with profound developmental or intellectual disability. Further, while sleep disturbance is common in this population, more extreme wake and sleep state dysregulation manifesting as cyclical insomnia and hypersomnia could be indicative of a mood disorder that may otherwise go undetected due to these obstacles to psychiatric evaluation. Careful attention to changes in affect during periods of prolonged wakefulness or periods of hypersomnia, as caregivers were able to observe in this case report, may prompt consideration of bipolar disorder.

Individuals with CDD demonstrating cyclic patterns of behavioral and sleep disturbance have been reported in the literature and observed within clinics at the CDKL5 Centers of Excellence, but they are not the norm as demonstrated in the review of sleep patterns in our Boston Children’s Hospital cohort. Mackay et al (6) identified an individual with CDD who experienced repetitive non-seizure episodes of somnolence followed by confusion, aggression, repetitive behavior and palilalia lasting 1 day to several months. She did not have a diagnosis of affective disorder.

We report an individual whose symptoms began in childhood and reached diagnostic criteria for bipolar disorder in adolescence. Her referral to psychiatry occurred shortly after her hypomanic episodes evolved to reach diagnostic criteria for bipolar disorder. Thus, there was not a significant delay in diagnosis of bipolar disorder, though it is possible that childhood episodes of irritability and somnolence may have represented undiagnosed depression. Delays and complications in the diagnostic process are, however, not uncommon amongst individuals with severe intellectual and developmental disabilities. Early studies purported that under-diagnoses of psychiatric disorders in individuals with ID could be a result of symptoms associated with ID obscuring psychiatric symptoms (diagnostic masking), or symptoms of psychiatric conditions being mistaken as symptoms of ID (diagnostic overshadowing) (11).

Despite the emergence of psychiatric diagnostic tools specific to ID such as the Diagnostic Manual – Intellectual Disability (first and second editions) in more recent years (12–14), individuals with ID are still often under-diagnosed with comorbid psychiatric disorders. One study found that in a cohort of 51 adults with severe/profound ID, more than half met criteria for a previously undiagnosed psychiatric disorder (15). A number of case reports and cohort studies also exist documenting lengthy and/or complicated diagnostic journeys for individuals with intellectual and/or developmental disability and symptoms of psychiatric disorders attributed to a variety of factors from patient’s communication abilities to systemic issues such as lack of provider experience, poor interdisciplinary collaboration, and high medical burden (6, 16–18). We suspect that diagnostic masking and overshadowing may be hindering the identification of other individuals with affective disorders in the CDD population. Communication and motor difficulties may be preventing observation of thought- and activity-related symptoms, while irritability, episodic changes in tone, and alternating periods of insomnia and somnolence may be non-specific or attributed to other features of CDD or medication side effects.

Though sleep dysregulation is common in CDD, the relationship between the reported individual’s dysregulated sleep and her mood led to clarification of this individual’s bipolar disorder diagnosis (2, 3). Reduced sleep is typically associated with worsened mood, irritability, and difficulty regulating negative emotions (19, 20). In the CDD population specifically, more severe sleep disturbances, notably insomnia and daytime sleepiness, have been reported to be associated with lower “negative emotions” scores on the QI-Disability measure, indicating reduced quality of life in that domain (4, 21). This domain includes some items that explicitly ask about mood (how often has the child become withdrawn with low mood) and others that approximate it based on caregiver observation of behavior (how often has the child been unsettled without apparent reason, how often has the child appeared upset or angry). In the setting of bipolar disorder, the association between sleep and mood occurs in reverse. Depressive episodes are typically associated with excessive sleep, and manic/hypomanic episodes with elevated mood are typically associated with a decreased need for sleep (22). This was the case for the individual we are reporting, with decrease in activity and increase in irritability occurring in tandem with episodes of hypersomnia while increased stimming, rocking, vocalizations, and giggling indicative of an elevated mood occur in tandem with a reduced need for sleep.

This individual highlights a need to consider psychiatric diagnoses in patients with developmental and epileptic encephalopathies such as CDD, including careful assessment of patterns of sleep and non-verbal expressions of mood. Further study of affective diagnoses in the CDD population is needed to clarify whether there is a specific association.

## Abbreviations

CDKL5: Cyclin dependent kinase like-5
CDD: CDKL5 Deficiency Disorder
ICDD: International CDKL5 Deficiency Disorder Database
ASD: Autism spectrum disorder
ADHD: Attention Deficit Hyperactivity Disorder
DSM: Diagnostic and Statistical Manual of Mental Disorders
ID: Intellectual Disability
